# Memory-effect based deconvolution microscopy for super-resolution imaging through scattering media

**DOI:** 10.1038/srep33558

**Published:** 2016-09-16

**Authors:** Eitan Edrei, Giuliano Scarcelli

**Affiliations:** 1 Fischell Department of Bioengineering, University of Maryland, College Park, MD USA

## Abstract

High-resolution imaging through turbid media is a fundamental challenge of optical sciences that has attracted a lot of attention in recent years for its wide range of potential applications. Here, we demonstrate that the resolution of imaging systems looking behind a highly scattering medium can be improved below the diffraction-limit. To achieve this, we demonstrate a novel microscopy technique enabled by the optical memory effect that uses a deconvolution image processing and thus it does not require iterative focusing, scanning or phase retrieval procedures. We show that this newly established ability of direct imaging through turbid media provides fundamental and practical advantages such as three-dimensional refocusing and unambiguous object reconstruction.

Imaging performances of traditional optical systems quickly degrade with increasing scattering^1^,^2^, so that in the diffusive regime only low resolution optical images can be obtained^3^,^4^. However, recently, novel strategies such as phase conjugation, scattering-matrix inversion, ultrasonic encoding or the so-called “memory effect” have revolutionized this field showing high resolution imaging and focusing behind turbid tissue up to several scattering lengths^5^,^6^,^7^,^8^,^9^,^10^,^11^,^12^,^13^,^14^,^15^,^16^,^17^,^18^,^19^. In many of these studies, the ability to image through turbid media is facilitated by additional information gathered on the scattering medium. For example, placing a “guide-star” in the object plane, iterative algorithms have been used to correct for the aberrations induced by the medium^10^; or the scattered field has been recorded and reversed to focus or scan the light back onto the object plane^20^,^21^. Here, we use the fundamental principles of the memory effect to enable direct wide-field imaging through turbid media using deconvolution image processing and a single-shot of the scattered intensity pattern. This enabled us to achieve, through turbid media, many of the same powerful features of standard deconvolution microscopy such as improved resolution as well as three-dimensional refocusing.

The memory-effect, first described in the 1980’s, is a peculiar phenomenon observed when light propagates through a scattering medium^22^,^23^. Within a certain range of impinging angles, known as the memory-effect angular range, the seemingly-random speckle patterns observed after the scattering medium are highly correlated. Deconvolution processing is a well-established and widely used technique in astronomy^24^, microscopy^25^ as well as non-optical imaging^26^. It relies on the linear properties of imaging systems for which the imperfect image recorded by an instrument can be written as the convolution of the perfect object function with the point spread function (PSF) of the optical system, i.e. its response to a point source. Measuring or estimating the PSF of the system, one can restore, i.e. deconvolve, a high quality image from the measured one^27^,^28^. The deconvolution operation is known to yield better resolution than the diffraction limit and significant improvements in image contrast thanks to the available information of the system’s response^29^.

While the power of the deconvolution operation has been known for decades and is ubiquitously used to correct for aberrations in optical systems, it has never been exploited for imaging through scattering media, because the PSF is known to present dramatic spatial variations due to scattering. However, we show here that when a turbid medium is within an imaging system, the correlation properties of the memory effect predict a spatially-invariant PSF within the memory-effect angular range. This is due to a fundamental property of the memory effect, i.e. that when a beam impinging upon a turbid medium is tilted, the generated speckle pattern acquires a linear phase gradient, hence it is only shifted but not distorted within the optical system. Thus, within the memory-effect range, deconvolution microscopy can effectively be used for high-quality image reconstruction through the scattering layer.

## Materials and Methods

A coherent laser beam generated by a solid state continuous wave laser (532 nm) was transmitted through a rapidly rotating ground glass resulting in an incoherent illumination. As objects we used a USAF chart (Thorlabs) or fabricated custom patterns with standard photolithography masks. A scattering medium (diffusive tape or ground glass 120 Grit, Thorlabs) was placed at 45 to 160 mm from the object, and a single lens (f = 250 mm, 60 mm) was used to image the plane of the object onto the camera (Mightex MCE-B013). A typical integration time for a single shot needed in our experiments was 20 ms. The PSF of the system was measured by replacing the object with an iris of typical size 10–50 μm. Deconvolution post-processing was performed by applying the Richardson-Lucy MATLAB based code to the raw data, no other image processing methods were used in this paper. The number of applied deconvolution iterations varied between 100 and 400.

## Results and Discussion

**Figure 1:**
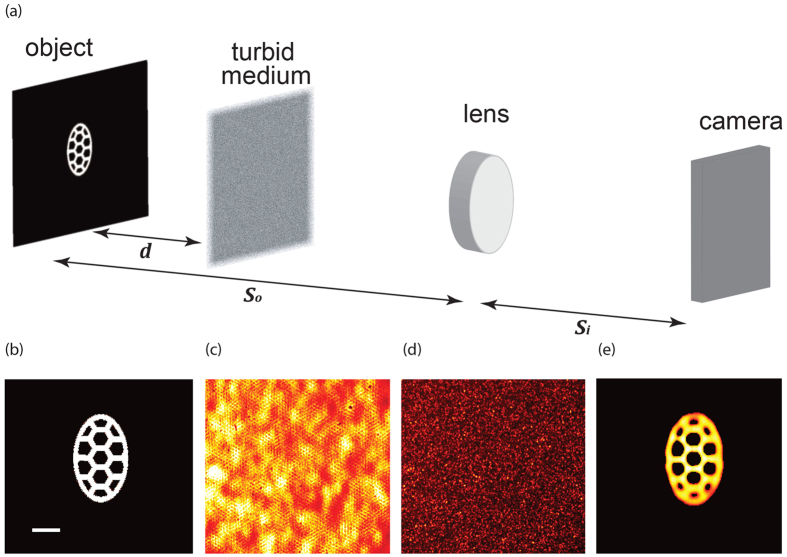
(**a**) Optical setup. A mask is placed a distance *d* = 16 *cm* behind a 120 grit ground glass diffuser. The plane of the object was imaged onto a CMOS camera using a 250 mm focal length lens. (**b**) Image of the object without turbid medium. Scale bar, 200 μm. (**c**) Scrambled image as recorded through the ground glass. (**d**) PSF of the overall setup recorded by replacing the object with an iris. (**e**) Reconstructed object after deconvolution.

We demonstrated our novel imaging technique experimentally with the setup in Fig. 1a. We illuminated an object aperture (e.g. Fig. 1b) with incoherent illumination, and imaged it onto a low-cost CMOS camera using a single-lens (f = 250 mm) imaging system aligned for 1.4 magnification. Within the imaging system, we placed a scattering medium (120-grit ground glass diffuser) so that the recorded image pattern was totally scrambled (Fig. 1c). We then applied a standard deconvolution operation (150 iterations of a Richardson-Lucy (R-L) algorithm^30^,^31^) using the PSF of the system measured by replacing the object with an iris of typical size 10–50 μm (Fig. 1d). This yielded an accurate reconstruction of the object hidden behind the scattering medium (Fig. 1e).

The performances of our imaging system can be analyzed using Huygens-Fresnel field propagation. The field at the image plane in the system in Fig. 1a can be written as:


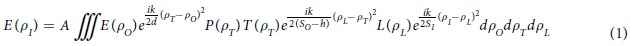


where 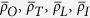 are the transverse coordinates in the plane of object, turbid medium, lens and image (camera) respectively. Using an explicit pupil function *P* as limiting aperture of the optical system, all the integrals can be performed on an infinite interval without loss of generality. We denoted the generic complex function of the turbid medium by *T*(*ρ*_*T*_), the lens operator by 
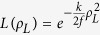
, and a proportionality constant by *A*. The distances from the object to turbid medium and lens are *d* and *S*_*O*_ respectively; the camera is located a distance *S*_*I*_ from the lens so that the Gaussian thin lens relation 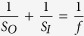 is followed.

Performing the integrals over the lens plane and applying the thin lens equation, Equation (1) simplifies to:


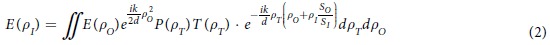


Recognizing the Fourier transform integral 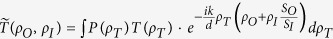
the intensity in the camera plane can be simplified to:


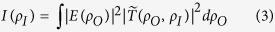


Thus the PSF of the system, obtained by using a delta function *δ*(*ρ*_*O*_ − *ρ*_*iris*_) in the object plane, is given by:


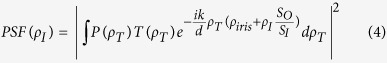


Therefore, the PSF of the system is the convolution of the pupil function of the imaging system with no turbid medium and the turbid medium function. With no turbid medium (i.e. T = 1), the system reduces to a standard imaging system with magnification 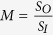. Importantly, it is clear from Equation 4 that a linear phase gradient acquired by *T*(*ρ*_*T*_), as the one occurring within the memory effect angular range, will yield a spatially-invariant PSF for the overall system.

**Figure 2:**
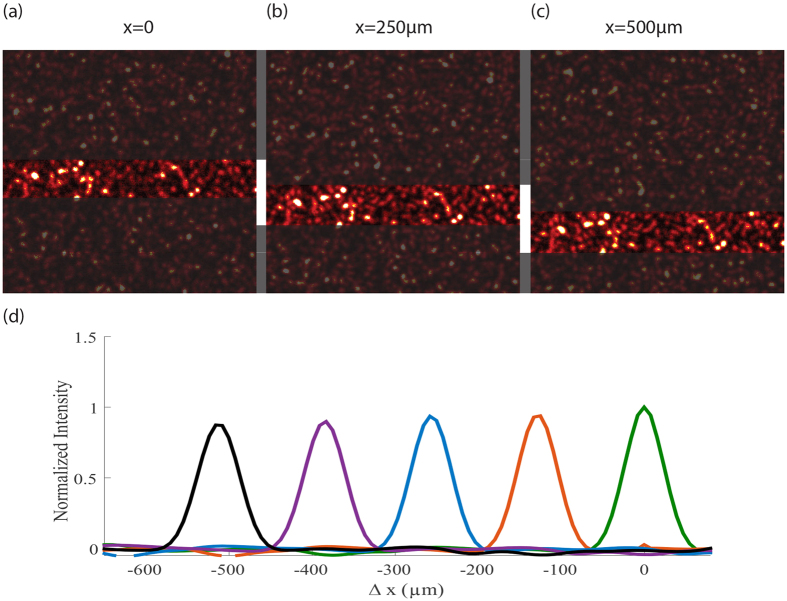
(**a–c**) PSF of the imaging system across the memory-effect field of view, measured by laterally shifting an iris in the object plane. The central area is highlighted to facilitate visualization of the undeformed shifted pattern. (**d**) Comparison of autocorrelation and cross correlations of different patterns at different iris positions demonstrating nearly unaltered PSFs within the memory-effect field-of-view.

To experimentally demonstrate the spatial invariance of the PSF, we replaced the object in the setup of Fig. 1a with a small iris and translated the iris laterally across the object plane behind a layer of opaque tape (50 um). Figure 2(a–c) show three representative images corresponding to three different locations of the iris. It is visually apparent that the PSF patterns are shifted but not distorted, as predicted. To quantify the PSF variation at different locations within our field of view we measured cross-correlations of the different patterns and compared it to their autocorrelation. As shown in Fig. 2(d), the height of the peak is nearly unaltered proving the invariance of the PSF within the angular range of the memory effect.

**Figure 3:**
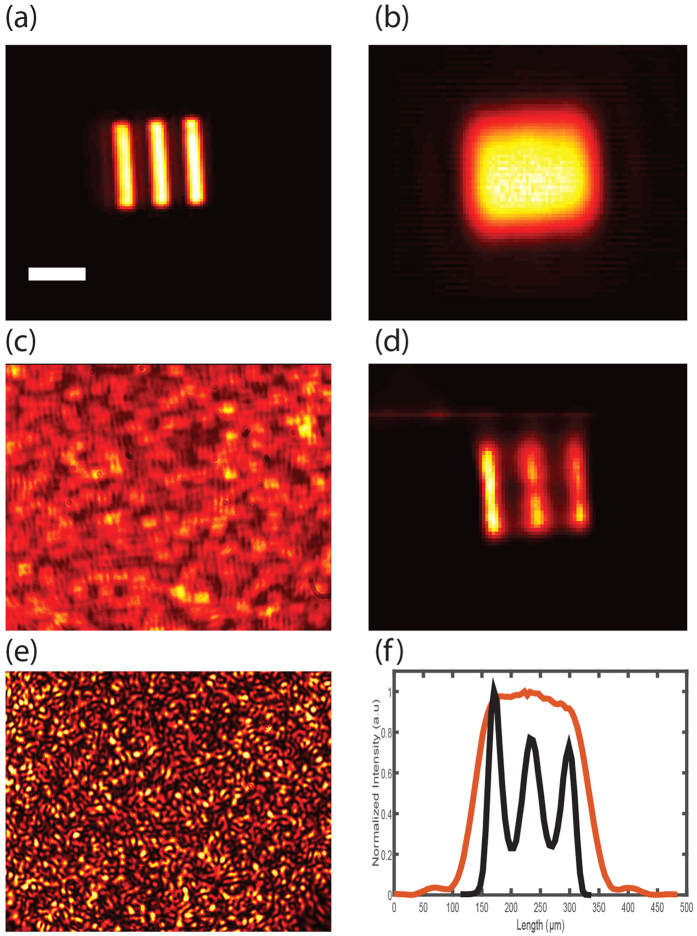
Super-resolution imaging through turbid medium. (**a**) Imaged object. Scale bar, 100 μm. (**b**) Blurred image of the object with no turbid medium and a standard imaging system of low numerical aperture. (**c**) Image of the object hidden behind a diffusive tape. (**d**) Image reconstruction by deconvolution. (**e**) PSF of the imaging system through the diffuser. (**f**) The intensity line plot obtained by averaging across (**b,d**) images visualizes the enhanced resolution of the reconstructed image (black) compared to standard imaging (red).

Next we show that deconvolution microscopy through turbid media retains the same powerful properties as if there were no turbid media such as resolution below the diffraction limit. For this, we used an object (Fig. 3a) whose features are smaller than the optical resolution of our imaging system as limited by the largest acceptance angle within the setup. In a standard configuration, i.e. with no turbid medium, the image appeared blurred due to the limited resolution of the imaging system (Fig. 3b). As we placed a diffusive tape in front of the object (d = 10 cm), the image appeared heavily scrambled (Fig. 3c). However, by deconvolving the scrambled image with the system PSF (in Fig. 3e), the image of the object was reconstructed and sharp (Fig. 3d). To better visualize the resolution improvement, in Fig. 3f, we show a line plot obtained by averaging across the standard image vs the reconstructed image via deconvolution through turbid medium. Using a point-like object, we have directly quantified that our protocol applying 150 R-L iterations yields a ~1.8-fold resolution improvement below diffraction limit consistent with previous demonstrations of resolution enhancement by deconvolution processing in standard optical systems^32^,^33^.

**Figure 4:**
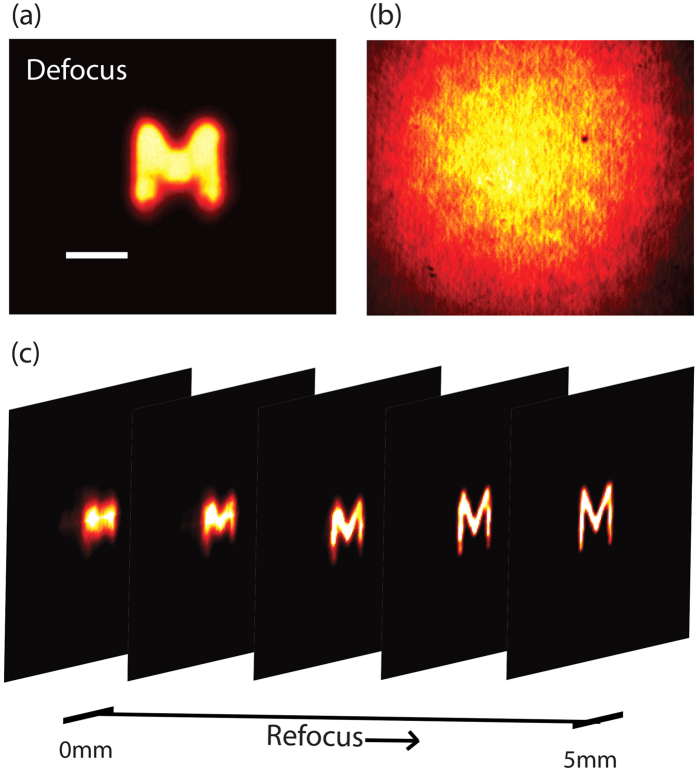
(**a**) Defocused image of an object as seen through an imaging system (with no turbid medium) focused on a plane 5 mm away from the location of the object. Scale bar, 200 μm. (**b**) The image is scrambled when a 120 grit ground glass diffuser is placed in front of the object. (**c**) Refocusing of the out-of-plane image through a turbid medium by deconvolution with the PSF measured at different axial locations.

Another important feature of deconvolution microscopy is its ability to re-focus out-of-plane images with results that in certain conditions can rival confocal microscopy^34^. This interesting feature is maintained in our protocol through turbid media and represents a marked difference over previous memory-effect methods characterized by extended depth of focus^7^. To demonstrate our reconstruction is sensitive to the axial location of the object we placed the object 5 mm away from the in-focus plane. In a standard imaging system without turbid medium, the image appeared blurred, as expected (Fig. 4a). The turbid medium placed at a distance d = 5 cm from the object scrambled the image completely (Fig. 4b). Next, we measured the three-dimensional PSF (3D-PSF) by placing an iris at different axial planes. By performing the deconvolution with the 3D-PSF function, we demonstrated successful reconstruction of a high quality image as well as 3D refocusing (Fig. 4c). Interestingly, also the decorrelation of the speckled PSF along the depth axis may introduce an optical sectioning effect^35^; however, this effect was not exploited in our experiment because in our setup the decorrelation full-width-at-half-maximum (FWHM) was 1.5 mm, five times larger than the depth of focus of the optical system (0.3 mm).

**Figure 5:**
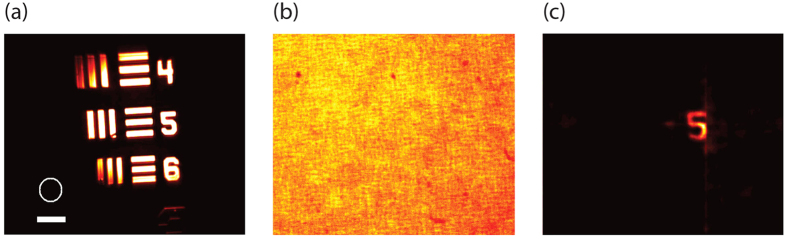
Large FOV illumination: (**a**) The object illuminated in the experiment (scale bar, 200 μm), the white circle indicates the FOV permitted by the memory effect (this experiment was performed using a 632 nm CW laser). (**b**) The blurred object as seen through a scattering layer of 20 tapes (total thickness of 1 mm). (**c**) The reconstruction of the object after applying 400 deconvolution iterations, only a small regime corresponding to the memory effect range was restored.

As in standard deconvolution procedures, noise is the practical limiting factor on the quality of the image reconstruction. When performing deconvolution imaging through turbid media based on memory effect, additional noise sources are introduced with respect to traditional free-space deconvolution imaging. For example, the components of the light emitted by the object at large angles will not be confined to the memory effect range and thus will result in an uncorrelated DC noise term. In our experiment, this noise term was minimized using a field stop within the optical system. Similarly, DC noise terms will be introduced if objects that are larger than the memory-effect field of view are illuminated, as they will produce uncorrelated patterns within the reconstruction procedure. To quantitatively analyze this effect, we recorded many uncorrelated speckle patterns (PSFs) produced by point sources placed at distant locations within the object plane. As expected, the deconvolution of each PSF with itself gives back a noiseless reconstruction of the individual point source; instead, as many uncorrelated patterns are added or simultaneously recorded, the signal to noise ratio (SNR) of the deconvolution reconstruction degrades. We quantified the SNR to remain greater than 1 with up to 40 uncorrelated patterns. This hints at an interesting feature of our protocol: deconvolution processing is robust against illumination outside the memory-effect field of view in the object plane, which instead is known to degrade phase-retrieval procedures. To demonstrate this feature experimentally, we used a turbid medium made of twenty 50 μm-thick layers of scattering tape and illuminated a large object placed 45 mm behind the medium (Fig. 5a). The field of view (FOV) of the memory effect (estimated at the FWHM) was 200 mdeg (corresponding to the white circle in Fig. 5a). Behind the turbid medium the object was completely blurred (Fig. 5b), as expected. After deconvolving the image with the measured PSF, we reconstructed the desired part of the object (Fig. 5c). While the illuminated area was 20 times larger than the one allowed by the memory effect, the reconstructed regime falls within the area dictated by the memory effect FOV.

Thus, we have demonstrated a new imaging approach that enables to perform imaging through turbid media below the diffraction limit of the optical system. As in other memory-effect based modalities, our new protocol is restricted in terms of scattering layers to go through by the angular memory effect range which is very limited in biological tissues^6^,^7^,^36^, however, those modalities have an infinite DOF and thus lack the ability to distinguish depth within a scene or an object^7^. Moreover, phase retrieval algorithms which are used in these studies impose strict limitations on the complexity of the objects that can be imaged and do not guarantee convergence to an unambiguous correct solution^37^,^38^; this is not a concern in our imaging protocol. However, it is important to note that in order to achieve these features, our protocol requires to have information about the PSF of the scattering medium. Thus, our technique is limited to situations where the turbid medium can be pre-characterized to infer the PSF or a guide-star within the medium can be measured. In this respect, techniques that pre-characterize the turbid medium have flourished in the past decade^39^. Measuring the transmission matrix to correct for sample induced aberrations as well as iterative focusing to obtain the desired shaped wave-front have been successfully demonstrated in highly scattering media^10^,^40^. However, these techniques generally have minutes-long acquisition times as they require either an iterative process or multiple acquisitions. A faster wave-front sensing and shaping modality is optical phase conjugation (OPC) in which the scattered light field is recorded and reversed to compensate for the aberrations of the medium^5^,^41^. Recently, the time needed for focusing light through scattering medium using OPC or digital OPC was reduced to several milliseconds^42^,^43^. However, for imaging purposes scanning across the object plane is often needed which increases the measurement time^20^,^21^. In contrast, here we reported a wide-field imaging modality where it is sufficient to record a single shot of the scattered intensity, and the image is obtained by deconvolution processing.

## Conclusion

In conclusion we have demonstrated that the fundamental principles of the optical memory effect can be effectively used to perform deconvolution microscopy of objects hidden behind visually opaque materials thus providing super-resolution imaging with three-dimensional refocusing capabilities. As we used a basic deconvolution algorithm, our technique is expected to significantly improve with more sophisticated deconvolution procedures^24^. Our imaging method can be extended to epi-detection configurations for practical applications by using established methods to estimate the system point spread function either by a blind process^44^ or using guide-stars embedded in the medium at the object plane^39^.

## Additional Information

**How to cite this article**: Edrei, E. and Scarcelli, G. Memory-effect based deconvolution microscopy for super-resolution imaging through scattering media. *Sci. Rep.* **6**, 33558; doi: 10.1038/srep33558 (2016).
